# Factors Related with Induced Abortion among Primigravid Women in Ho Chi Minh City, Vietnam

**DOI:** 10.2188/jea.12.375

**Published:** 2007-11-30

**Authors:** Nguyen Thi Diem Van, Nguyen Quang Vinh, Trinh Huu Phuc, Huynh Nguyen Khanh Trang, Tran Mong Thuy, Nguyen Thi Tu Van, Nguyen Duy Phong, Tran Thi Loi, Aya Goto

**Affiliations:** 1 Department of Obstetrics and Gynecology, University of Medicine and Pharmacy, Ho Chi Minh City.; 2 Tu Du Obstetrical and Gynecological Hospital.; 3 Department of Infectious Diseases, University of Medicine and Pharmacy, Ho Chi Minh City.; 4 Population Council, Vietnam, and Department of Public Health, Fukushima Medical University, School of Medicine.

**Keywords:** induced abortion, contraception, Vietnam

## Abstract

A case-control study was conducted between July and August 2001 in Ho Chi Minh City to investigate factors associated with having induced abortions among primigravid women aged 16 to 38 years. Interviews were conducted with 87 women undergoing abortion (cases) and 81 pregnant women coming for antenatal care (controls). Multiple logistic regression analysis revealed that older age (odds ratio [OR]=0.84) and being married (OR=0.05) decreased the risk of getting unintended pregnancy leading to abortion. Risk factors of obtaining an abortion were not being exposed to family planning promotion on television (OR=2.28) and not knowing the adverse effects of abortion (OR=10.26). Descriptive analysis of contraceptive behavior showed that 41% of cases had never used contraceptives and the reason for non-usage was lack of knowledge of any type of contraceptives in 43% of the cases. Additionally, only 24% of cases had discussed about family planning with their partners. Effective contraceptive methods should be promoted among young population, both females and males, and they need to be informed of the consequences of abortion. Television programs might be an effective mode of providing the information.

While abortion rates have declined sharply in socialist countries in central and eastern Europe and former Russian countries, abortion rate in Vietnam remains high, and the number of abortions in the public sector was 1.52 million in 1996.^1^ Vietnam has implemented two-child policy and recommended induced abortion and intrauterine devices as family planning methods allowing the maximum state control. With the vigorous family planning program, the Demographic Health Survey in 1997 reported that 55.8% of married women were using modern contraceptives, among which intrauterine devices were the most commonly used method.^2^ However, contraceptive choice is limited and one survey estimated that the proportion of women with unmet need for family planning was about 25%.^3^ Consequently, Vietnam has become the country with extremely high abortion rate; a Vietnamese woman has about 2.5 abortions during her reproductive years.^2^

As for the characteristics of women undergoing abortion, a number of previous studies pointed out that abortion seekers tend to be single, young, poor, and with lower level education, and previous pregnancy history including live births and abortions.^4^^,^^5^ On the other hand, a study in the United Kingdom concluded that there is no difference between women who are having abortions and those who are not.^6^ In Vietnam, there have been few quantitative researches on abortion seekers, especially nulliparous women whose fertility can be affected by complications of abortion.

Ho Chi Minh City is one of the largest cities in Vietnam with more than five million residents. Urbanization has skirted the city quickly and many immigrants have come from other provinces, even from provinces in the northern part of the country. Along with the city’s development, life style of youth has modernized. They live more independently from their family, freer in interrelating with lovers,^7^ and are able to earn money to meet their demand. Despite their worries about the consequences of their sexual behaviors and efforts to obtain information to prevent unintended pregnancy, they often end up exposing themselves to the risks of illegal abortion services.

City mass media, in the forms of newspapers, publications, radio, television, and other electronic communications, disseminate family planning information. However, the number of abortions performed daily in the city hospitals proves that these information are not effective and sufficient. The number of the abortions in Ho Chi Minh City was 1.32 million in 2000 according to the Center for Mother and Child Protection.^8^ At Tu Du Hospital alone, there were about 30 thousand abortions in the same year, representing more than 20% of legal abortions in the city. For the prevention of unwanted pregnancies leading to abortion and protection of young women’s fertility, this study aims to investigate characteristics of primigravid abortion seekers and their contraceptive knowledge.

## METHODS

An unmatched case-control study was conducted in Ho Chi Minh City from July 23 through August 2, 2001. Survey was conducted at a clinic of Midwife School and Tu Du Obstetrical and Gynecological Hospital, both of which are affiliated institutes of University of Medicine and Pharmacy, Ho Chi Minh City. We managed the ratio of subjects from the Midwife School and Tu Du Hospital to be one, both for cases and controls.

Cases were primigravid women attending family planning clinic for termination of pregnancy, and controls were primigravid pregnant women attending prenatal care. Due to lack of systematic registration and sufficient data collection at hospitals in Vietnam, we were able to obtain only limited information on our study population. The numbers of abortion and antenatal care clients in 2001 were 29,867 and 100,163 at Tu Du Hospital and 6,488 and 14,731 at the clinic, respectively. The proportion of primigravidae among them was unknown.

Our study subjects were a convenient sample. Chief-nurses of the two study places first checked the reproductive history of all abortion and antenatal care clients they encountered during the study period. Then, only the primigravid women were sent to interviewers to have the study procedures explained. In spite of this non-random sampling, it is unlikely that chief-nurses gave preference to women sharing certain characteristics over others. Subjects who gave an oral consent were interviewed.

We used a structured questionnaire, which consisted of 30 questions about socio-demographic background, reproductive history, and knowledge on contraception. One interview was approximately 30 to 45 minutes. Prior to the main survey, a pilot study was conducted in the same setting to revise the questionnaire and to confirm the feasibility of the survey.

Trained midwife students conducted 171 face-to-face interviews. The maximum care was paid to standardize the interview procedure. The questionnaire was formed in a way that interviewers just needed to read the questions, and they were instructed not to paraphrase the questions. Interviewers had a training session prior to the survey and the research team explained an interview manual. Mid-term review sessions were held two times during the survey period to discuss any problems that occurred during the interview. In addition, two lecturers of the mid-wife school were assigned to supervise the students, and the assigned lecturers and the research team monitored the interviews periodically.

Data were analyzed using the statistical software STATA version 6 for Windows (STATA Corporation, College Station, Texas). Descriptive analysis of reproductive behavior were conducted both for cases and controls. Multiple logistic regression analysis was applied to examine the factors associated with abortion.

The variables included in the analyses are listed in Tables 1, 2, and 3. They were chosen from factors previously reported to have relationship with seeking abortion. Continuous variables (ages of women and their partners, and age at first sexual intercourse) and dichotomous variables were directly put into the models, while variables with more than two categories were re-classified to dichotomies.

**Table 1.** Results of univariate analysis showing association of abortion with socio-demographic, health behavioral and reproductive factors

Variables		Number (%) or median [min, max]		Odds	

		Cases (n= 87)	Controls (n=81)	ratio (95% CI)	
Age (years) ^a^		22 (16, 36)	25 (19, 38)	0.80	(0.72-0.88)
Residence					
	In Ho Chi Minh City (no)	14(16)	11(14)	1.22	(0.51-2.86)
	Duration of residence in Ho Chi Minh City (years) ^a^	11 [ 0, 31]	13 [ 1, 35]	0.97	(0.94-1.00)
Education (higher than secondary level)		20(23)	23(28)	0.75	(0.37-1.50)
Having own income (yes)		22(25)	24(30)	0.80	(0.40-1.58)
Exposure to media					
	Read newspapers or magazines (not everyday)	46(53)	29(36)	2.01	(1.08-3.73)
	Listen to radio (not everyday)	51(59)	50(62)	0.87	(0.47-1.63)
	Watch television (not everyday)	23(26)	10(12)	2.55	(1.12-5.76)
Social activities					
	Christian (no)	70(80)	62(77)	1.26	(0.60-2.64)
	Member of a youth, women or trade union (no)	56(64)	57(70)	0.76	(0.39-1.45)
Family circumstances					
	Living with husband (no)	43(49)	6(7)	12.21	(4.81-31.01)
	Marital status (married)	49(56)	79(98)	0.03	(0.00-0.14)
Age at first sexual intercourse^a^		21[15,34]	24[17,37]	0.80	(0.72-0.89)
Partner					
	Age (years) ^a^	26 [17, 56]	29 [19, 43]	0.89	(0.83-0.95)
	Age difference (partner’s age - woman’s age) ^a^	4 [-8, 26]	4 [-2, 14]	0.99	(0.93-1.06)
	Know partner’s education level (no)	11(13)	2(3)	5.64	(1.21-26.31)
	Education (higher than secondary level)	21(28)	28(36)	0.68	(0.34-1.35)
	Know partner’s drinking habit (no)	8(9)	1(1)	8.10	(0.99-66.28)
	Drinking habit (not everyday)	75(95)	79(99)	0.23	(0.02-2.17)

CI: confidence interval

a: Odds ratios are for one unit change in the variables.

**Table 2.** Results of univariate analysis showing association of abortion with contraceptive knowledge

Variables		Number (%)		Odds	

		Cases (n= 87)	Controls (n=81)	ratio (95% CI)	
Obtained family planning information recently from:				
	Radio (no)	70(80)	59(74)	1.46 (0.70-3.03)
	Television (no)	46(53)	22(28)	2.95 (1.54-5.64)
	Papers and magazines (no)	41(47)	38(48)	0.98 (0.53-1.80)
	Source other than mass media (no)	74(85)	72(90)	0.63 (0.24-1.61)
Knowledge about condom				
	Heard about condom (no)	5(6)	3(4)	1.56 (0.36-6.77)
	Know that condom can prevent pregnancy (no)	20(23)	19(23)	0.97 (0.47-1.99)
	Know that condoms have to be put on an erected penis (no)	61(70)	43(53)	2.07 (1.10-3.90)
Knowledge about oral contraceptives (OC)				
	Heard about OC (no)	9(10)	9(11)	0.91 (0.34-2.42)
	Know that OC has to be taken everyday (no)	35(40)	35(43)	0.88 (0.47-1.63)
	Know when to start OC in menstrual cycle (no)	50(57)	51(63)	0.79 (0.42-1.47)
Knowledge about abortion				
	Know that abortion is not a method of contraception (no)	18(21)	8(10)	2.38 (0.97-5.82)
	Know that abortion may have adverse effects on health (no)	7(8)	1(1)	7.00 (0.84-58.20)

CI: confidence interval.

**Table 3.** Results of multivariate analysis showing factors associated with abortion

Variables	Odds ratio (95% CI)	
Age (years) ^a^	0.84	(0.75-0.94)
Marital status (married)	0.05	(0.01-0.23)
Obtained family planning information recently from television (no)	2.28	(1.05-4.93)
Know that abortion may have adverse health effects (no)	10.26	(0.92-113.41)

CI: confidence interval.

a: Odds ratio is for one unit change in the variable.

Univariate analysis was first conducted for each item. Items that showed significant or borderline significant association with abortion (p< 0.05 and p<0.1, respectively) were put into multivariate analysis. Among items that showed a strong significant correlation (correlation coefficient > 0.5, p < 0.05) or that were close in their contents, variables that fitted better to the model or the ones with more specific meaning were chosen for multivariate analysis. Adequacy of the final model was tested using goodness-of-fit test.

As to minimize misclassification in the analysis stage, we included a question to assess pregnancy intention. The cases and controls were stratified into two groups: one group with intended pregnancy and another with unintended pregnancy. Then, the same analysis was repeated using the cases whose pregnancies were unintended (pure cases) and the controls whose pregnancies were intended (pure controls).

## RESULTS

Despite the sensitive nature of several questions, there were only 2 refusals out of 173 women we approached and the proportions of missing answers were less than 1% for all items in the questionnaire. Three observations were eliminated from the analysis due to misprinting of questionnaires. Data of 168 subjects, 87 cases (43 from Tu Du Hospital and 44 from the clinic) and 81 controls (39 from Tu Du Hospital and 42 from the clinic), were analyzed.

Univariate analyses showed that the cases were different from the controls in many socio-demographic characteristics (Table 1). Women who were seeking abortion were younger than their controls; the median age of cases was 22 years, whereas that of controls was 25 years. Other items showing difference between cases and controls were exposure to media (newspapers or magazines and television), cohabitation with partner, marital status, age at first intercourse, age of partner, knowing partner’s education level, and drinking habit.

A large part of our questionnaire was shared to questions assessing contraceptive knowledge (Table 2). Among the sources of family planning information, only television showed a significant difference between the two groups. Compared with controls, higher proportion of cases did not know when to put on condoms, considered abortion as a contraceptive method, and did not recognize the adverse health effects of abortion.

Table 3 shows the results of multivariate analysis. Older age (odds ratio [OR]=0.84) and being married (OR=0.05) decreased the risk of getting unintended pregnancy leading to abortion. Risk factors of obtaining an abortion were not being exposed to family planning programs on television (OR=2.28), and not knowing the adverse effects of abortion (OR=10.26).

The proportions of women with unintended pregnancy among abortion seekers (pure cases) and those with intended pregnancy among antenatal care patients (pure controls) were 84% and 78%, respectively (Table 4). The same analyses were applied to the pure case and control groups, and similar results were obtained, except that a statistical significance diminished for the knowledge about adverse effects of abortion.

**Table 4.** Contraceptive behaviors of cases and controls

Variables		Number (%)	

		Cases (n= 87)	Controls (n=81)
Frequency of intercourse just before this pregnancy			
	At least once per week	27(31)	41(51)
	Less than once per week	60(69)	40(49)
Pregnancy intention			
	Intended	14(16)	63(78)
	Unintended	73(84)	18(22)
Contraceptive usage around the time of conception			
	Yes	51(59)	21(26)
	No	36(41)	60(74)
Contraceptive method used around the time of conception^a^			
	Oral contraceptives	11(22)	5(24)
	Condom	10(20)	8(38)
	Emergency pill	7(14)	0(0)
	Rhythm method	12(24)	5(24)
	Coitus interrupt	7(14)	0(0)
	Other methods	3(6)	3(14)
Reason for not using contraceptives^a^			
	Wanted to become pregnant	5(14)	48(81)
	Did not know any contraceptive method	15(43)	5(8)
	Other reasons	15(43)	6(10)
Discussed about family planning with:^b^			
	Partner	21(24)	29(36)
	Mother	6(7)	4(5)
	Friends	17(20)	16(20)
	Nobody	47(54)	39(48)
Discussed about this pregnancy with:^b^			
	Partner	69(79)	68(84)
	Mother	11(13)	30(37)
	Friends	6(7)	12(15)
	Nobody	6(7)	6(7)

a: These are the proportions among subjects who used or not used contraceptives around the time of conception.

b: These questions are multiple choice and the percentages in column do not add up to 100%.

Controls, who wanted to become pregnant, included higher proportion of women having intercourse at least once per week; 51% for controls and 31% for cases (Table 4). It was noted that 41% of cases were not using contraceptives around the time of conception. The reason for the non-usage was lack of knowledge on any contraceptives in 43% of the cases. Even for cases who had ever used contraceptives, proportions of those who used the oral contraceptives, condom, and emergency pill were only 22%, 20% and 14%, respectively, and those of unreliable traditional methods like a rhythm method and coitus interrupt were as high as 24% and 14%. Additionally, more cases had never discussed about family planning with anyone; 54% for cases and 48% for controls, and only 24% of cases had discussed with their partners.

## DISCUSSION

The multivariate analysis revealed that the risk factors of abortion among primigravidae were being single and young, not knowing side effects of abortion, and insufficient exposure to family planning information through television. In addition, usage of effective contraceptives and the communication with partners in sexual relationship were found to be insufficient among the abortion seekers through the descriptive analysis.

There are two unique advantages in our study; one is that we examined our findings with pure cases and controls, and the other is that we selected primigravidae as our subjects. Women are known to have ambiguous feeling toward pregnancy planning and the decision making process to determine whether to give birth or abort is complex.^9^^,^^10^ Some intended pregnancies end in abortion and some unintended pregnancies in birth because of medical and socio-economic reasons. Our findings were confirmed with pure cases and controls after eliminating the potential misclassification.

The first abortion is the most critical event for every woman considering its effects on her physical and mental health. We targeted primigravid women since their pregnancy and abortion experiences have substantial influences over their following fertility. An abortion causes long term complications, and the abortion may lead to another abortion as well. One study among young single abortion cases in Hanoi reported that 23% were repeaters.^11^ Other abortion statistics from Nghe An reported that abortion clients with a history of one or more abortion(s) in the past accounted for 47%.^12^ If we identify the factors associated with abortion seeking behavior among primigravid women and reflect the findings in abortion prevention strategies, we will be able to reduce the abortion rates among women of all reproductive age groups and the rate of physical complications of the procedure.

There is another technical advantage for selecting primigravidae. Previous case-control studies aiming to investigate characteristics of abortion seekers suffered from a misclassification of cases and controls, because controls might have been women with a history of past abortions. To solve this problem, one study from Italy excluded women having undergone abortion in the past three years from the control group.^13^ Unfortunately, this additional exclusion criterion caused a difficulty in collecting the sufficient number of controls. In our study, we targeted only primigravidae to avoid the misclassification and the necessity of setting such a problematic exclusion criterion.

To our knowledge, our study is the first one in Vietnam to identify being young and single as the factors related to abortion seeking behavior. Abortion surveillances in other countries showed that the abortion seekers were typically young and unmarried.^14^^,^^15^^,^^16^ One analysis of Vietnamese abortion statistics reported that abortions concentrated among married women in their peak childbearing ages, but it was predicted that more single young women might become sexually active with modernization.^17^ Qualitative studies among Vietnamese young women undergoing abortion found that the low contraceptive use among them was due to poor family planning knowledge and a misconception that contraception was for married couples.^11^^,^^18^

It was interesting to find out the ignorance of adverse effects of abortion as a risk factor, considering the historical and political background regarding abortion in Vietnam.^19^ When the fertility control was one of the first priorities of the country’s development strategies in the 1980s and the early 1990s, availability of contraceptives was still limited. Government policy and health worker’s interest and knowledge were biased in favor of intrauterine devices, female sterilization, and abortion.^17^^,^^20^ The clandestine abortion was blamed to be life threatening, and the quality of abortion service and its access were improved. Abortion has become the main activity of every family planning clinic, and in a short period of time, the total fertility rate in Vietnam decreased from 3.8 children in 1989 to 2.7 in 1996 according to Vietnamese census data.^21^ Abortion has been utilized as a method of contraception and consequently, adverse effects of the procedure have not been sufficiently disseminated.

Another interesting risk factor found to relate with abortion in our study was the less exposure to contraceptive information on television. Similar results were found in India and Bangladesh; contraceptive practice was significantly associated with the increase in the exposure to different forms of contraceptive information through media, particularly through television.^22^ Moreover, a study in Nigeria among secondary school students identified television as the main source of knowledge about reproductive health.^23^ The question still remains if the significant relationship between contraceptive information on television and abortion is confounded by income. Even with some variation in income, our subjects shared the same financial level that enabled them to obtain health services at two major health institutes in Ho Chi Minh City. In addition, as many as 87.7% of households in the city have television sets, which are affordable media devices for fairly wide range of population.^8^ The confounding by income thus is less likely to be a major problem.

In many areas in the world, advertisements of contraceptive products on television in prime time are now accepted by the public, and are gaining success as evidenced by increased sales of several marketers.^24^ With the high popularity of television in Ho Chi Minh City, messages about accurate information and specific instructions on family planning could be sent even among the poorly educated and low incomes. Unfortunately, due to lack of professional staff and funding for the Information, Education and Communication campaigns at the television station, programs on contraceptives are limited to simple lectures given during office hours. The content of the programs is confined to intrauterine devices and oral contraceptives for the pregnancy prevention and condoms for the sexually transmitted disease prevention. Financial as well as technical supports from governmental and non-governmental agencies are needed to overcome the weaknesses and shortcomings of the current programs on family planning in Ho Chi Minh City.

It should be paid attention that 41% of abortion cases in our study had never used contraceptives, and that the major reason for the non-usage was lack of knowledge of any types of contraceptives. For those who had ever used a contraceptive method, not many used the effective methods like condoms, oral contraceptives, and emergency pills, which are recommended for young population. Strong resistance from society is expected when promoting contraceptives among teenagers and young adults.^25^ However, 24% of our subjects, including cases and controls, were single, and age at first sexual intercourse was under 20 years for 18% (data not shown). Young people’s unmet need for contraception should be recognized and reversible modem contraceptives need to be more advertised.

Descriptive analysis of contraceptive behavior indicated little partner involvement in family planning. Although as much as 80% of cases discussed with their partners about the present pregnancy, most had not discussed about contraception before becoming pregnant. Furthermore, only a little over 10% used condoms, which is a male contraceptive method. In Thailand, condom use has become popular with the implementation of the “100% condom program” and proven to be effective in limiting HIV/AIDS.^26^ On the other hand, China has the highest contraceptive prevalence rate in the world, yet the abortion rate is still increasing due to insufficient contraceptive knowledge and men’s refusal to practice contraception.^27^ Taking the lessons learned from other countries, the Vietnamese National Committee for Population and Family Planning has recommended strategies to increase male participation in the reproductive health programs since 1998. Despite the government policy, a study in 1999 reported that Vietnamese men, who were the primary decision-makers, considered women to be responsible for family planning.^28^ The Information, Education and Communication materials for changing attitudes and behavior toward family planning should target both women and men.

Our survey had four potential limitations. The first one is a misclassification bias, which we attempted to reduce by choosing primigravidae as explained above. Second, there might be a recall bias since we did not limit the gestational age of cases and controls. Most of the abortion cases came to hospital before 8th gestational week, but the pregnancy of antenatal care patients were more advanced and might recall less about the exposure to contraceptive information through mass media around the time they became pregnant. Even with this potential bias, it was found that the cases were less exposed to the contraceptive information on television than the controls. Third, there was a potential observer bias. We minimized this bias by masking interviewers from knowing the study design, dividing them into 2 groups; one group for cases and the other for controls, and by conducting analysis only after the completion of data collection. As described in the method section, both interview groups were well trained and a questionnaire was carefully developed in order to avoid any differences in the ways questions were asked between the two groups. The last limitation of our study is related to the sensitiveness of the topic itself. We tried to overcome this issue by giving careful consideration on the way to ask questions and the privacy of women during interview. As a result, we only had a very few non-respondents and missings.

In conclusion, our survey provides local authorities with the evidence of factors associated with abortion in Ho Chi Minh City. The findings suggest that effective contraceptive methods should be promoted among young population, both females and males, and that they need to be informed of the potential adverse effects of abortion. Well-structured and attractive television programs could provide all of this information.
